# Adjacent thoracic lymph node metastases originating from two separate primary cancers: case report

**DOI:** 10.1186/1477-7800-5-22

**Published:** 2008-10-02

**Authors:** Khalid A El-Gendy, Gary K Atkin, Robert E Brightwell, Paul Richman, Jeremy I Livingstone

**Affiliations:** 1 Department of Surgery, Watford General Hospital, Vicarage Rd, Watford, WD18 0HB, Hertfordshire, UK; 2 Department of Biosurgery and Surgical Technology, Imperial College London, London, UK; 3 Department of Histopathology, Mount Vernon Hospital, Rickmansworth Rd, Northwood, Middlesex, HA6 2RN, UK

## Abstract

Reported is an unusual case of adjacent thoracic lymph nodes demonstrating metastases from two different primary malignancies. A 51 year-old woman with a previous history of bilateral breast cancer underwent a radical gastro-oesophagectomy for adenocarcinoma of the lower third of the oesophagus. The resection specimen demonstrated breast and oesophageal metastases in adjacent thoracic lymph nodes. Mechanisms for this phenomenon, including the known local immune suppression on lymphoid cells by oesophageal carcinoma cells, are discussed.

## Background

Oesophageal carcinoma cells have been shown to exert a local immune suppression on regional lymphoid cells [1], facilitating the metastatic deposit and survival of cancer cells from a distant malignancy [2]. Reported is an unusual case of adjacent thoracic lymph nodes demonstrating metastases from two different primary malignancies. As far as we know this is the first report of such a phenomenon, and the finding parallels the previously reported 'collision phenomenon' in which two distinct primary carcinomas metastasise to the same lymph node.

## Case presentation

A 51 year-old woman was diagnosed with adenocarcinoma of the distal third of the oesophagus after presenting with dysphagia. She had a past history of recurrent bilateral breast cancer necessitating bilateral mastectomy and several courses of radiotherapy to both breasts as well as the left axilla and left chest wall. She had subsequently undergone bilateral breast augmentations with silicone implants.

Preoperative staging CT for the oesophageal malignancy did not reveal any enlarged abdominal or thoracic lymph nodes, or any other evidence of metastases, and so she underwent radical surgery by subtotal oesophagectomy. At laparotomy, there were no liver or peritoneal metastases and a radical resection was performed incorporating abdominal and mediastinal lymph node clearances and a stapled oesphago-gastric anastomosis at the level of the azygous vein. Post-operatively she developed pneumonia and she remained in the intensive care unit for 32 days. She was subsequently discharged after a hospital admission of 63 days. Three months later she developed a skin metastasis within the right thoracotomy scar, and she died soon after.

**Figure 1:**
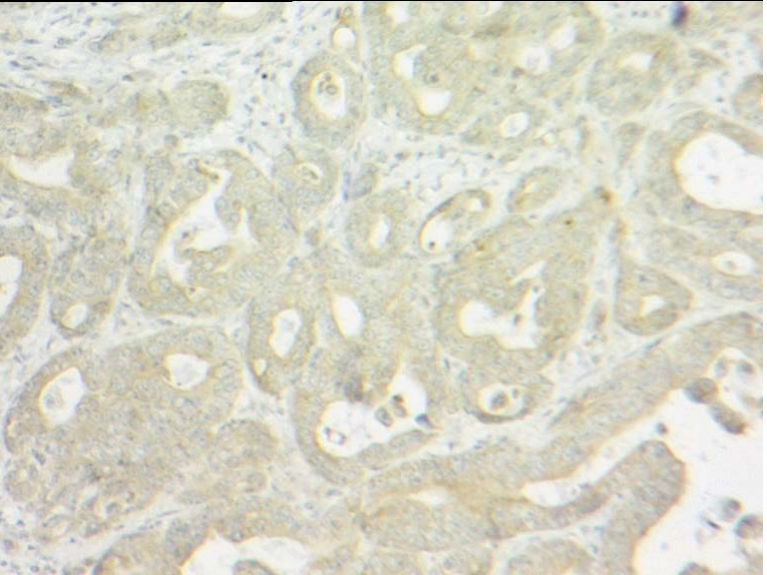
Oesophageal metastasis in paraoesophageal lymph node staining negatively for oestrogen receptor (magnification × 100).

**Figure 2:**
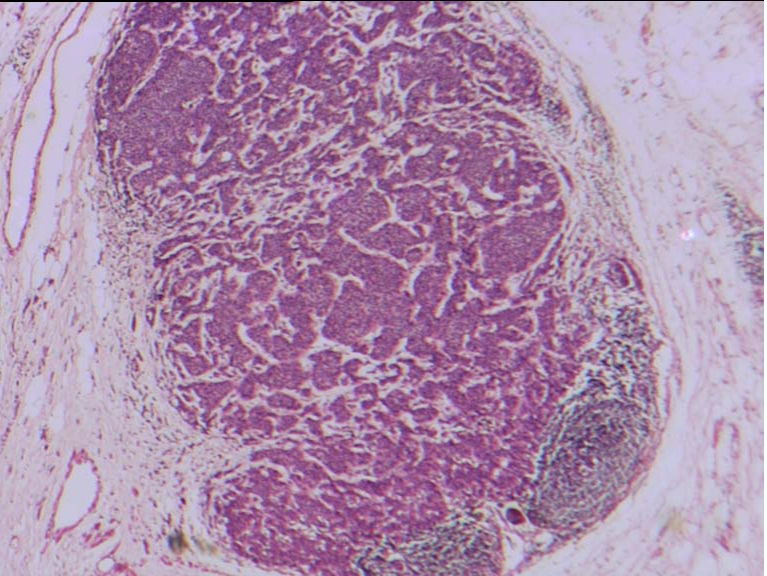
Paraoesophageal lymph node containing metastatic ductal breast carcinoma (magnification × 100).

**Figure 3:**
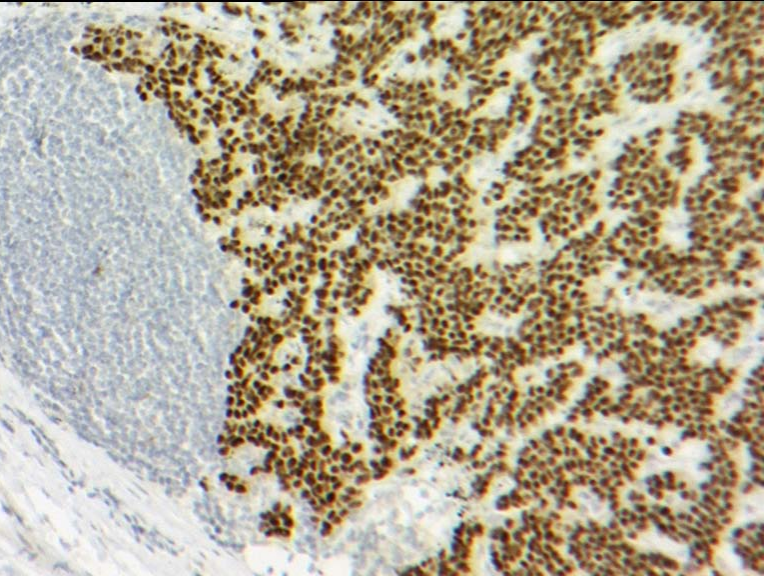
Lymph node metastasis demonstrating oestrogen receptor-expressing breast carcinoma (magnification × 100).

**Figure 4:**
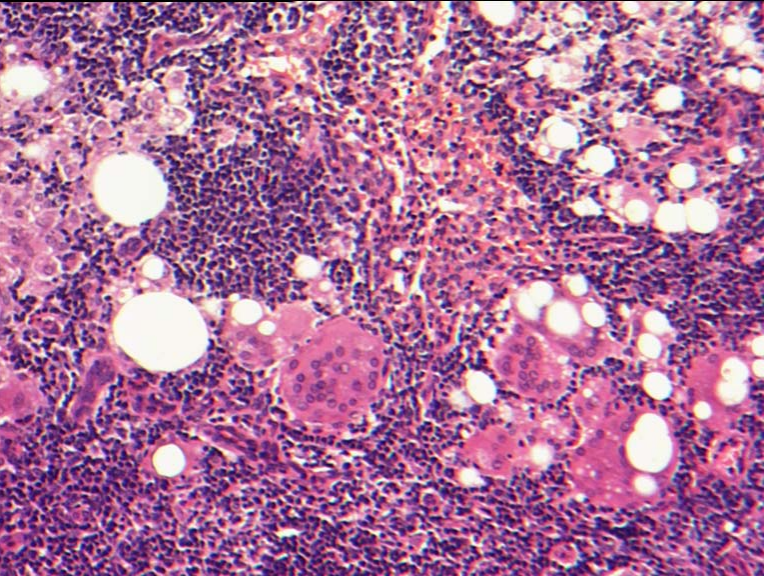
Paraoesophageal lymph node demonstrating silicone-associated reaction (magnification × 100).

Histological analysis of the oesophagectomy specimen revealed a poorly differentiated adenocarcinoma at the gastro-oesophageal junction. Tumour infiltrated through the muscularis propria into serosa and adjacent fat. Two paraoesophageal lymph nodes contained adenocarcinoma with appearances compatible with metastases from the oesophageal primary, and which on immunohistochemistry were positive for the epithelial marker cytokeratin 7 (CK7) but negative for oestrogen receptor [Figure 1] and gross cystic disease fluid protein-15 (GCDFP-15), both of which are breast carcinoma related molecules. Two further, adjacent lymph nodes contained carcinoma compatible with metastatic ductal carcinoma of breast origin [Figure 2]. These expressed oestrogen receptors [Figure 3] and gross cystic disease fluid protein-15 (GCDFP-15). Several lymph nodes not involved by tumour demonstrated a silicone-associated reaction [Figure 4].

## Conclusion

The present case demonstrates adjacent thoracic lymph node metastases from two distinct primary malignancies. Immunohistochemical profiling was used to determine the site of origin for each metastasis. Two lymph nodes were positive for GCDFP-15, a highly specific marker for metastatic breast carcinoma with a specificity and positive predictive value of 99% [3], whereas the oesophageal metastases expressed the marker CK7 and were negative for the breast carcinoma related molecules. This rare case is, to the authors' knowledge, the first report of mixed metastatic disease involving metastases from two distinct primary cancers occurring within adjacent lymph nodes. Metastasis within a single lymph node from two distinct primary cancers has previously been documented. This is termed the 'collision phenomenon' and has been seen with bladder and prostate, prostate and colon, urothelial and prostate, and prostate and gastric cancers [4-8]. All of these reported cases used immunohistochemical characterisation of the lymph node to confirm the tumour of origin.

Thoracic lymph node metastasis from a primary breast cancer is an uncommon occurrence [9], and several mechanisms may be postulated to explain the present case, as well as the collision phenomenon. Regional immune suppression by oesophageal squamous carcinoma has been reported [1] and O'Mahony *et al *[10] proposed the existence of a soluble factor released by oesophageal tumour cells which induces apoptosis in lymphoid cells. It may be, therefore, that a local immune suppression exerted by the oesophageal malignancy facilitated the metastatic deposit and survival of cancer cells from a distant breast malignancy. In vitro work, however, suggests this mechanism may be active only for squamous carcinoma of the oesophagus, as a suppression of regional immune function by oesophageal adenocarcinoma cells was not seen [10]. Other mechanisms facilitating regional metastases from distant cancers include the role of tumour infiltrating lymphocytes, which have been shown to have a poor cytotoxic effect on tumour cells compared with standard lymphocytes [11]. In the present case, there was no radiological evidence of thoracic lymph node disease demonstrated throughout the follow-up of the breast cancer. However, breast cancer is often considered as a systemic disease [12] and it may be that development of an oesophageal malignancy subsequently led to the deposit of circulating breast carcinoma cells within an adjacent thoracic lymph node. However, it may have been that the thoracic lymph node metastases were present initially and were too small for detection by CT.

An increased risk of oesophageal cancer has been seen in breast cancer patients treated with radiotherapy [13-16]. Squamous cell carcinomas are most commonly reported, but breast cancer patients treated with radiotherapy are four times more likely to suffer from oesophageal adenocarcinoma compared to those not treated with radiotherapy [15]. The risk is greatest ten years or more following radiotherapy and there is no increased risk in breast cancer patients not receiving radiotherapy [15,16].

Another pathological point of interest in the present case was the silicone-associated reaction evident in several thoracic lymph nodes. Silicone dissemination is well recognised following insertion of silicone prostheses, and has been reported in blood and lymphatic tissue as well as tissues adjacent to the prosthesis [17]. Leakage of silicone after breast augmentation has been implicated in the development of conditions such as Kikuchi's disease (histiocytic necrotising lymphadenitis), as well as systemic disorders such as autoimmune and connective tissue disease [17]. Debate continues over the oncological safety of silicone implants following early reports of breast adenocarcinoma and squamous cell carcinoma in patients with previous silicone breast augmentation [18]. Uncertainty over the safety of silicone implants led to the Food and Drug Administration (FDA) restricting their use in the United States in 1992. Recent studies with long term follow up have failed to demonstrate a link with the development of breast cancer, however, and the FDA's position may be revised in the near future.

## Consent

Verbal informed consent was obtained from the patient in the presence of 3 of the authors prior to death for the publication of this case report and any accompanying images. The patient was unable to provide written consent due to illness and there is no available next of kin from which we could obtain written consent. This has been discussed and approved with the Editor-in-Chief.

## Competing interests

The authors declare that they have no competing interests.

## Authors' contributions

All authors have read and approved the final manuscript. KE, GA, and RB all contributed significantly towards article generation and subsequent revision. PR provided the histological figures and JL oversaw the project and offered critical analysis.
